# Insight into Neutrophil Extracellular Traps through Systematic Evaluation of Citrullination and Peptidylarginine Deiminases

**DOI:** 10.1155/2019/2160192

**Published:** 2019-03-12

**Authors:** Caitlyn L. Holmes, Daeun Shim, John Kernien, Chad J. Johnson, Jeniel E. Nett, Miriam A. Shelef

**Affiliations:** ^1^Department of Medicine, University of Wisconsin-Madison, 1685 Highland Avenue, Madison, WI 53705, USA; ^2^Department of Pathology and Laboratory Medicine, University of Wisconsin-Madison, 1685 Highland Avenue, Madison, WI 53705, USA; ^3^Department of Medical Microbiology and Immunology, University of Wisconsin-Madison, 1550 Linden Drive, Madison, WI 53706, USA; ^4^William S. Middleton Memorial Veterans Hospital, 2500 Overlook Terrace, Madison, WI 53705, USA

## Abstract

In rheumatoid arthritis, an autoimmune inflammatory arthritis, citrullinated proteins are targeted by autoantibodies and thus thought to drive disease. Neutrophil extracellular traps (NETs) are a source of citrullinated proteins and are increased in rheumatoid arthritis and therefore also implicated in disease pathogenesis. However, not all NETs are citrullinated. One theory aiming to clarify the intersection of citrullination, NETs, and rheumatoid arthritis suggests that specific stimuli induce different types of NETs defined by citrullination status. However, most studies do not evaluate uncitrullinated NETs, only citrullinated or total NETs. Further, the requirement for peptidylarginine deiminase (PAD) 2 and 4, two important citrullinating enzymes in neutrophils and rheumatoid arthritis, in the formation of different NETs has not been clearly defined. To determine if specific stimulants induce citrullinated or uncitrullinated NETs and if those structures require PAD2 or PAD4, human and murine neutrophils, including from PAD4^−/−^ and PAD2^−/−^ mice, were stimulated *in vitro* and NETs imaged and quantified. In humans, phorbol myristate acetate (PMA), ionomycin, monosodium urate (MSU), and *Candida albicans* induced NETs with MSU and *C. albicans* inducing primarily citrullinated, PMA primarily uncitrullinated, and ionomycin a mix of NETs. Only ionomycin and *C. albicans* were strong inducers of NETs in mice with ionomycin-induced NETs mostly citrullinated and *C. albicans*-induced NETs a mix of citrullinated and uncitrullinated. Interestingly, no stimulus induced exclusively citrullinated or uncitrullinated NETs. Further, PAD4 was required for citrullinated NETs only, whereas PAD2 was not required for either NET in mice. Therefore, specific stimuli induce varying proportions of both citrullinated and uncitrullinated NETs with different requirements for PAD4. These findings highlight the complexity of NET formation and the need to further define the mechanisms by which different NETs form and their implications for autoimmune disease.

## 1. Introduction

Neutrophil extracellular traps (NETs) are complex webs of chromatin and proteins extruded from neutrophils during the programmed cell death process of NETosis [1]. NETs can be antimicrobial [1–4] and aid in the resolution of inflammation [5]. However, NETs also appear to be pathologic in multiple autoimmune diseases including rheumatoid arthritis [6, 7], systemic lupus erythematosus [8, 9], antiphospholipid antibody syndrome [10, 11], and small vessel vasculitis [12]. In rheumatoid arthritis, the pathology is thought to hinge on the presence of citrullinated proteins on NETs. Citrullination is the posttranslational deimination of arginine residues to citrullines, catalyzed by the peptidylarginine deiminases (PADs). Most patients with rheumatoid arthritis generate autoantibodies that bind citrullinated proteins [13]. Since NETs are increased in rheumatoid arthritis [6, 7] and contain citrullinated proteins targeted by anti-citrullinated protein antibodies [6, 14, 15], NETs are hypothesized to be a significant source of citrullinated proteins in rheumatoid arthritis, thus driving inflammation.

However, different stimuli can produce NETs with different composition and cargo [6, 16, 17] as well as potentially different types of NETs with different roles for citrullination [18]. For example, leukotoxic hypercitrullination (LTH) generates NETs characterized by hypercitrullination and can be induced by the membrane attack complex [19] or pore-forming bacterial proteins [20]. In contrast, phorbol myristate acetate (PMA) stimulates NETosis without citrullination [16, 17]. Based on the literature, a categorization of NETs has been hypothesized with NETosis induced by several stimuli including PMA, fungi, and monosodium urate (MSU) without citrullination and LTH induced by pore-forming molecules with citrullination [18]. Such a categorization is helpful for understanding different types of NETs, their mechanisms of formation, their functions, and their potentially different roles in autoimmune disease. For example, if LTH induced by the membrane attack complex leads to hypercitrullination and NETosis induced by *Candida albicans* does not involve citrullination, then membrane attack complex-induced LTH might drive rheumatoid arthritis and *C. albicans*-induced NETosis might not. However, there is variation among reports regarding which stimuli induce NETs. For example, some studies show that ionomycin and *C. albicans* induce extensive NETs and others report that these stimuli induce few to no NETs [3, 16, 21–25]. Further, most studies evaluate either total or citrullinated NETs, so much less is known about uncitrullinated NETs. Given the gaps in the literature and the importance of understanding different types of NETs in autoimmune disease, it would be of benefit to determine which stimuli induce citrullinated and uncitrullinated NETs.

There are also questions regarding the roles of PAD2 and PAD4 in the formation of different types of NETs. These two PADs are found in neutrophils [26] and the rheumatoid joint [27] and each independently contributes to murine rheumatoid arthritis [28, 29]. Further, specific inhibitors of each of these PAD enzymes are being developed with consideration for treatment in rheumatoid arthritis [30, 31]. Many NET studies have focused on PAD4, which citrullinates histones enhancing chromatin decondensation during NETosis [32, 33]. Further, PAD4 was shown to be required for the production of NETs induced by various stimuli [2, 28, 34–38]. However, PMA inhibits PAD4 while inducing NET formation [16] and PAD4 is not required for NETs formed in response to *Klebsiella pneumoniae* [39] or *C. albicans* [25], suggesting that PAD4 may not be required for the formation of all NETs. Much less is known about the role of PAD2 in NETosis. PAD2 is present on NETs [40], but is not required for the formation of NETs in response to TNF*α* and LPS [28]. No other studies have investigated a requirement for PAD2 in NET formation, a problematic gap in knowledge since PAD2 appears to be required for the bulk of citrullination in a murine model of rheumatoid arthritis [28].

In this report, we systematically quantify murine and human NETs formed in response to ionomycin, PMA, MSU, and *C. albicans* and determine if they are citrullinated or uncitrullinated. We also evaluate if PAD2 or PAD4 is required for the NETs induced by these stimuli.

## 2. Materials and Methods

### 2.1. Human Subjects

This study was carried out in accordance with the recommendations of the Association for the Accreditation of Human Research Protection Program. The protocol was approved by the Institutional Review Board at the University of Wisconsin-Madison. All subjects gave written informed consent in accordance with the Declaration of Helsinki. Human subjects 18 years or older were recruited and provided a blood sample.

### 2.2. Animals

Age- and sex-matched wild-type, PAD2^−/−^ [41], and PAD4^−/−^ [2] mice back-crossed to a DBA/1J background (Jackson Laboratories, Bar Harbor, USA) were used. Animals were housed in a pathogen-free facility. This study was carried out in accordance with the principles of the Basel Declaration and recommendations of the ARRIVE guidelines, the National Centre for the Replacement, Refinement and Reduction of Animals in Research. The protocol was approved by the University of Wisconsin Animal Care and Use Committee.

### 2.3. Purification and Stimulation of Human Neutrophils

The human blood was collected into EDTA tubes, and neutrophils were purified using the EasySep Direct Neutrophil Isolation Kit (StemCell Technologies, Vancouver, Canada) according to the manufacturer's protocol. Neutrophil purity was at least 95% by flow cytometry. Neutrophils were plated onto acid-washed, poly-L-lysine (Sigma Diagnostics, Livonia, USA) coated 12 mm glass coverslips at a concentration of 50,000 cells per coverslip in media containing RPMI 1640 (Thermo Fisher Scientific, Waltham, USA) with 2% fetal bovine serum (Atlanta Biologicals, Flowery Branch, USA) and 1% penicillin-streptomycin solution (Corning, Tewksbury, USA). Neutrophils were treated with the following and incubated for 4 hours at 37°C, 5% CO_2_: 4 *μ*M ionomycin (MilliporeSigma, Darmstadt, Germany), 560 *μ*g/mL MSU crystals (InvivoGen, San Diego, USA), 25 nM PMA (Fisher BioReagents, Waltham, USA), or 1 × 10^6^ *Candida albicans* strain SC5314 [42].

### 2.4. Purification and Stimulation of Murine Neutrophils

The mouse femurs and tibias were flushed with the media described above, and neutrophils were purified with the EasySep Mouse Neutrophil Enrichment Kit (StemCell Technologies) according to the manufacturer's protocol. Neutrophil purity was at least 91% by flow cytometry. Neutrophils were plated onto acid-washed, poly-L-lysine-coated 12 mm glass coverslips at a concentration of 70,000 cells per coverslip in the media described above. Neutrophils were incubated for 4 hours at 37°C, 5% CO_2_ with the following stimuli: 5 *μ*M ionomycin, 1200 *μ*g/mL MSU crystals, 25 nM PMA, or 1 × 10^6^ *C. albicans* strain SC5314.

### 2.5. *Candida albicans*

*C. albicans* was prepared as previously described [28]. Briefly, *C. albicans* was stored in 15% glycerol stock at -80°C with yeast extract peptone dextrose (YPD) medium supplemented with uridine (1% yeast extract, 2% peptone, 2% dextrose medium, and 0.08% uridine) prior to the experiments. Single *C. albicans* colonies were grown overnight in YPD with uridine at 30°C and orbital shaking at 200 RPM. Planktonic cells were used by diluting cultures 20-fold and incubating and shaking for an additional 2 hours. *C. albicans* was centrifuged and washed twice with the final concentration adjusted to 4 × 10^7^ cells/mL in phosphate-buffered saline (PBS) before use.

### 2.6. Immunofluorescence

After stimulation, neutrophils were processed as previously [28] for immunofluorescence. Cells were incubated for 30 minutes at 4°C with 4% paraformaldehyde, 1% NP-40, and 0.5% Triton X-100 in PBS and then washed with PBS. Coverslips were then blocked overnight with 2.5% bovine serum albumin (BSA), 5% goat serum, and 0.5% Tween-20 in PBS followed by staining for 1 hour with anti-citrulline IgM (F95, MilliporeSigma) diluted 1 : 200 in blocking solution, washing with PBS, and then incubating for 1 hour with anti-mouse IgM-TRITC (SouthernBiotech, Birmingham, USA) diluted 1 : 200 and 4′,6-diamidino-2-phenylindole (DAPI) (Sigma-Aldrich, St. Louis, USA) diluted 1 : 1000 in blocking solution, and washing with PBS. Coverslips were mounted on glass microscope slides with Aquamount (Thermo Fisher Scientific). All staining was performed at room temperature. A Leica Fluorescence Microscope with Image Pro-Plus v.6.3 (Media Cybernetics, Rockville, USA) was used to image five predetermined fields on the coverslip at 400x. For Supplementary Figures, processing, staining, and imaging were identical as above, but F95 was replaced by anti-histone H4, citrulline 3 (MilliporeSigma) and anti-mouse IgM-TRITC was exchanged for anti-rabbit IgG-TRITC (Jackson Laboratories).

### 2.7. Quantification of NETs

Neutrophils and NETs present in the five predetermined fields were counted by eye in a blinded manner. NETs were defined as neutrophils with significant enlargement of the DNA area beyond the size of a condensed nucleus (evident in unstimulated samples) with spread morphology and diffuse DNA structure [43]. Citrullinated NETs also stained positively with F95.

### 2.8. Statistics

A *t*-test was used to compare the percentage of NETs between untreated neutrophils and each stimulant as well as between wild-type and PAD-deficient neutrophils. A *p*value < 0.05 was considered significant.

## 3. Results

**Figure 1:**
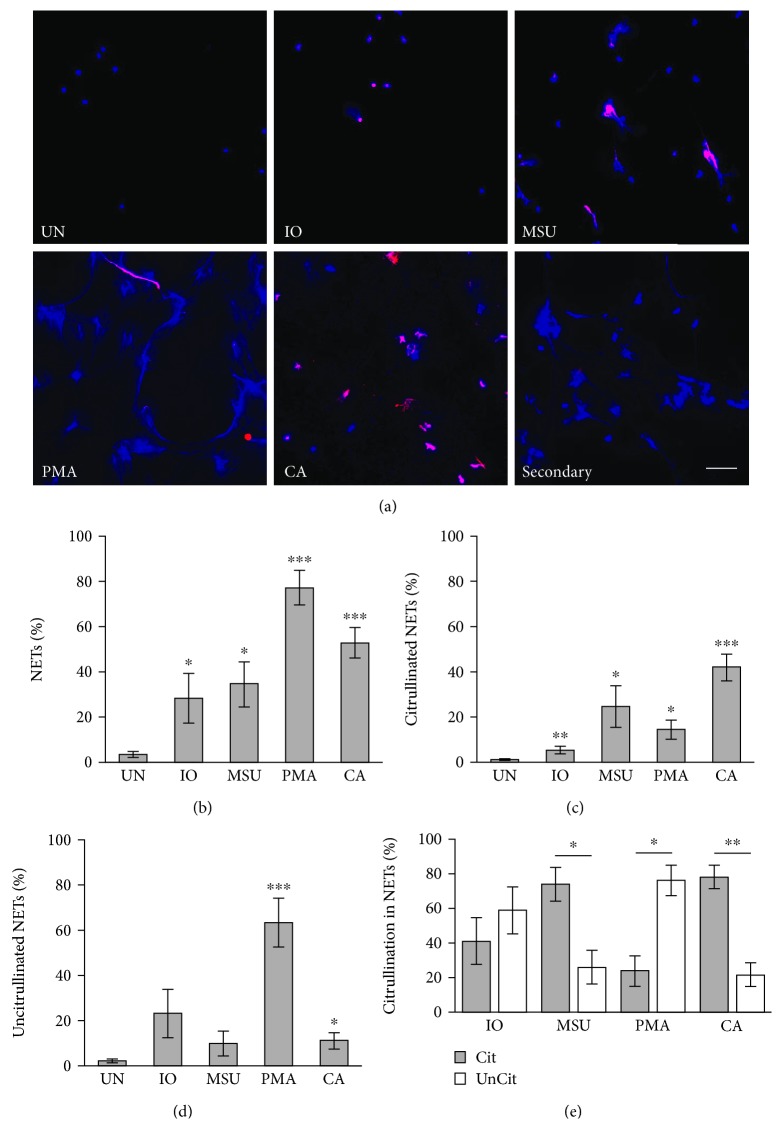
Induction of NETs in human neutrophils. Human neutrophils were left untreated (UN) or were treated with ionomycin (IO), MSU, PMA, or *C. albicans* (CA), fixed, and stained with DAPI (blue) and anti-citrulline antibody (pink). Image labeled “Secondary” was created by stimulating neutrophils with *C. albicans* and staining without the F95 primary antibody and only the anti-mouse IgM-TRITC secondary antibody as a negative control. (a) Representative images at 400x, scale bar = 50 *μ*M. The number of neutrophils and NETs were quantified. Graphs depict the average and SEM for percent of neutrophils that formed total NETs (b), citrullinated NETs (c), and uncitrullinated NETs (d) for each condition with percent NETs for each stimulant compared to untreated. (e) The percent of citrullinated versus uncitrullinated NETs was compared for each stimulus with average and SEM graphed. For all panels: *n* = 9; ^∗^ *p* < 0.05, ^∗∗^ *p* < 0.01, and ^∗∗∗^ *p* < 0.001.

Ionomycin (a calcium ionophore and pore-forming molecule), MSU crystals (which activate leukocytes via Toll-like receptors and the inflammasome driving gout), PMA (which activates protein kinase C and thus NF-*κ*B), and *C. albicans* are diverse and common stimulants of NETosis with innumerable connections to autoimmune disease. To determine if these stimulants induce citrullinated and/or uncitrullinated NETs, human neutrophils were isolated from peripheral blood and incubated with no treatment or each stimulant for 4 hours followed by fixation, staining to detect DNA and citrullinated proteins, imaging, and quantification. As expected and as confirmation of a lack of stimulation upon purification, untreated neutrophils generated almost no NETs (Figures 1(a) and 1(b)). Ionomycin, MSU, PMA, and *C. albicans* all induced more NETs than untreated neutrophils (Figures 1(a) and 1(b)). As shown in Figure 1(e), MSU and *C. albicans* induced primarily citrullinated NETs, whereas PMA induced mostly uncitrullinated NETs. However, PMA also induced some citrullinated NETs, more than unstimulated neutrophils (Figure 1(c)). *C. albicans* induced more citrullinated NETs as well as more uncitrullinated NETs than untreated neutrophils (Figures 1(c) and 1(d)). The ionomycin-induced NETs were a mix of citrullinated and uncitrullinated (Figure 1(e)). Given the low levels of citrullination at 4 hours after PMA or ionomycin treatment, we also quantified NETs 8 and 20 hours after PMA or ionomycin treatment. Similar numbers of citrullinated NETs were seen at those time points as compared to the 4-hour time point (data not shown). Finally, because F95 may cross-react with homocitrulline, we repeated our experiments quantifying citrullinated NETs using an antibody against citrullinated histone H4. The numbers of citrullinated NETs and uncitrullinated NETs were similar using this antibody (Figures 1(c) and 1(d) versus Supplementary Figure 2B, C), although in general slightly fewer citrullinated and slightly more uncitrullinated NETs were detected for each condition leading to some differences in the ratio of citrullinated versus uncitrullinated NETs (Figure 1(e) versus Supplementary Figure 2D).

**Figure 2:**
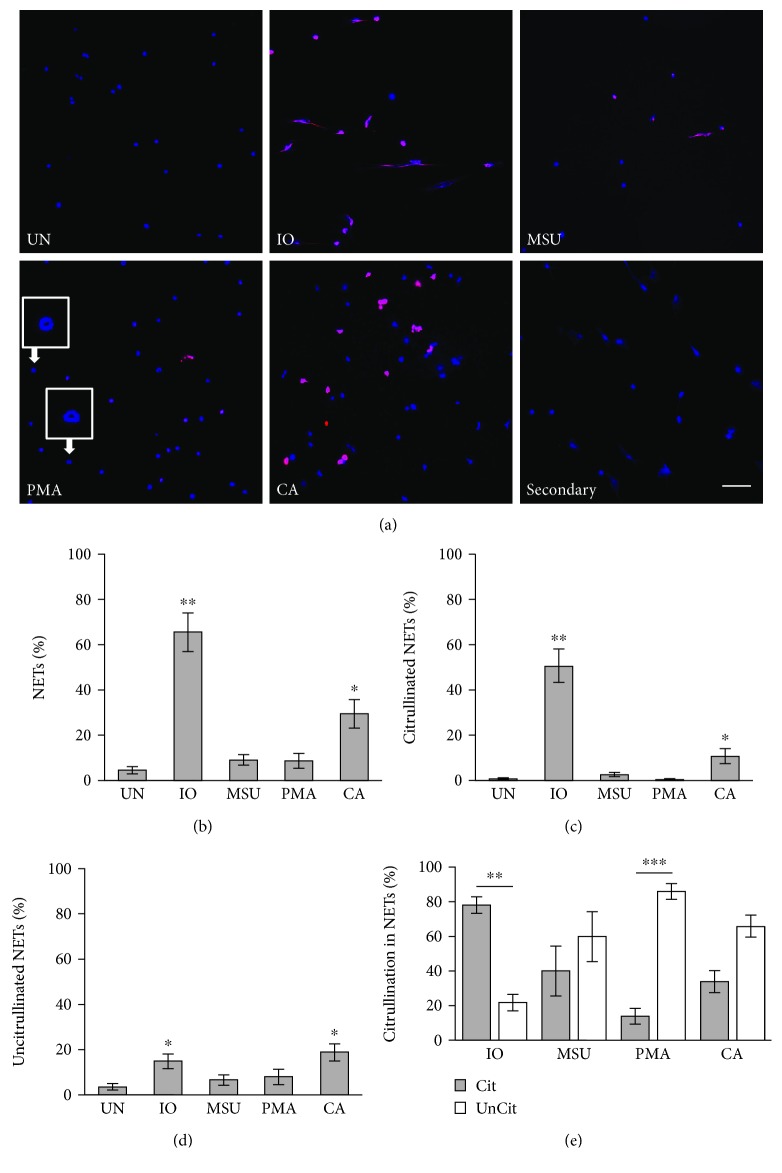
Induction of NETs in murine neutrophils. Murine neutrophils were left untreated (UN) or were treated with ionomycin (IO), MSU, PMA, and *C. albicans* (CA), fixed, and stained with DAPI (blue) and anti-citrulline antibody (pink). Image labeled “Secondary” was created by stimulating neutrophils with ionomycin and staining without the F95 primary antibody and only the anti-mouse IgM-TRITC secondary antibody as a negative control. (a) Representative images at 400x, scale bar = 50 *μ*M. Enlarged insets demonstrate donut-like structures (doNETs). The number of neutrophils and NETs were quantified. Graphs depict the average and SEM for percent of neutrophils that formed total NETs (b), citrullinated NETs (c), and uncitrullinated NETs (d) for each condition with percent NETs for each stimulant compared to untreated. (e) The percent of citrullinated versus uncitrullinated NETs was compared for each stimulus with average and SEM graphed. For all panels: *n* = 6; ^∗^ *p* < 0.05, ^∗∗^ *p* < 0.01, and ^∗∗∗^ *p* < 0.001.

Since mice are commonly used as an experimental model for autoimmune diseases involving NETs, we wanted to determine if findings would be similar in mice. We purified neutrophils from murine bone marrow and induced and quantified NETs as above. Unlike in human neutrophils, ionomycin was a very strong inducer of murine NETs and these structures were primarily citrullinated (Figures 2(b) and 2(e)). Also unlike in humans, MSU induced NETs variably in mice and murine MSU-induced NETs were a mix of citrullinated and uncitrullinated structures (Figures 2(b) and 2(e)). With murine neutrophils, PMA did not induce more NETs than untreated neutrophils (Figure 2(b)), which is also different than human neutrophils. Even when we stimulated with tenfold higher concentrations of PMA, similar results were seen (data not shown). However, like human neutrophils, the few NETs that were induced by PMA were uncitrullinated (Figure 2(e)). Also, PMA, and no other stimulant, led to the formation of ring-shaped structures of variable size in about 30% of murine neutrophils. Human neutrophils did not commonly make these structures as visualized by immunofluorescence, but similar structures could be seen by electron microscopy (Supplementary Figure 1) and perhaps could be called “doNETs” given their donut-like shape. As in human neutrophils, *C. albicans* was a strong inducer of NETs (Figure 2(b)) and *C. albicans* induced a mixture of citrullinated and uncitrullinated NETs (Figure 2(e)). Of note, both human and murine *C. albicans-*induced NETs were smaller in size compared to NETs induced by other stimuli. Finally, like human neutrophils, similar proportions of citrullinated and uncitrullinated NETs were seen with activation for 8 and 20 hours as compared to 4 hours (data not shown). Results for F95 agreed with results for anti-citrullinated histone H4 with very small differences seen only for PMA likely due to the low number of NETs with this condition (Supplementary Figure 3).

**Figure 3:**
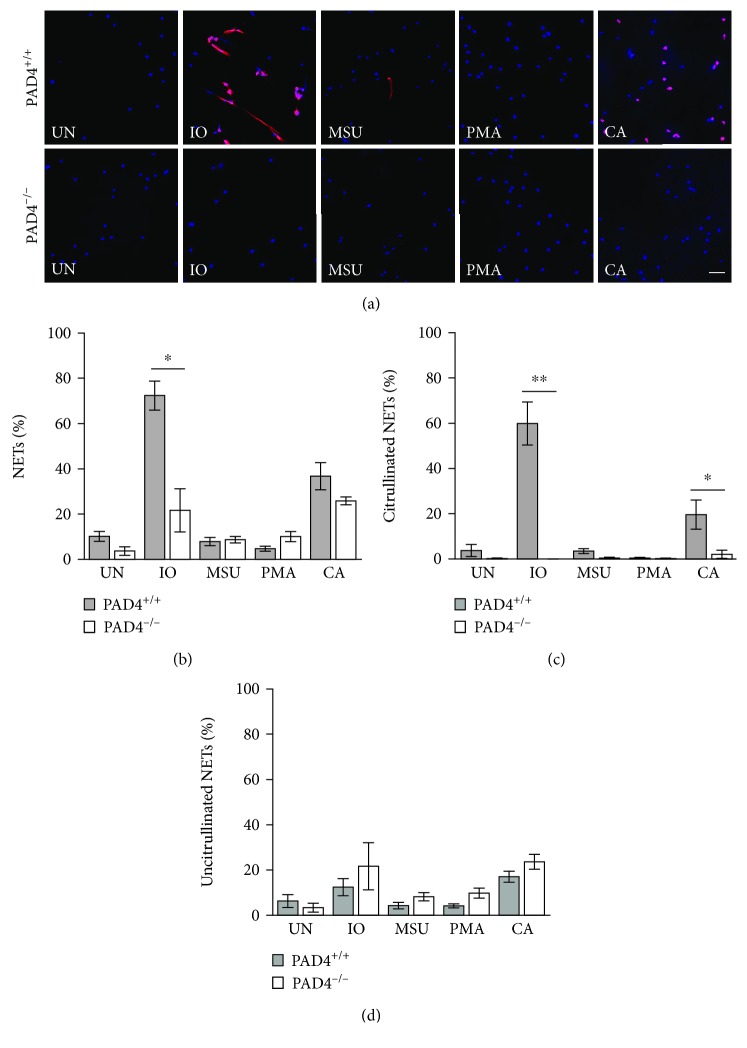
PAD4 is required for the formation of citrullinated NETs in murine neutrophils. Bone marrow neutrophils from PAD4^+/+^ and PAD4^−/−^ mice were left untreated (UN) or were treated with ionomycin (IO), MSU, PMA, and *C. albicans* (CA), fixed, and stained with DAPI (blue) and anti-citrulline antibody (pink). (a) Representative images at 400x, scale bar = 50 *μ*M. The number of neutrophils and NETs were quantified. Graphs depict the average and SEM for percent of neutrophils that formed total NETs (b), citrullinated NETs (c), and uncitrullinated NETs (d) for each condition with percent NETs for each stimulant compared between PAD4^+/+^ and PAD4^−/−^ mice. For all panels: *n* = 4; ^∗^ *p* < 0.05 and ^∗∗^ *p* < 0.01.

**Figure 4:**
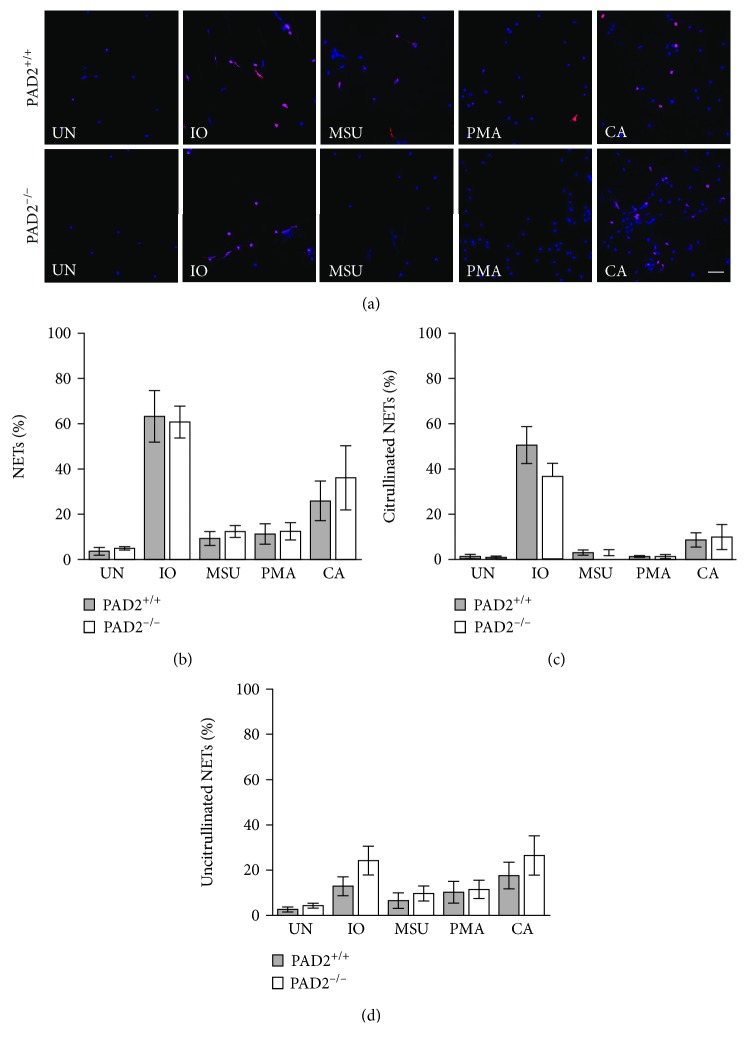
PAD2 is not required for the formation of NETs in murine neutrophils. Bone marrow neutrophils from PAD2^+/+^ and PAD2^−/−^ mice were left untreated (UN) or were treated with ionomycin (IO), MSU, PMA, and *C. albicans* (CA), fixed, and stained with DAPI (blue) and anti-citrulline antibody (pink). (a) Representative images at 400x, scale bar = 50 *μ*M. The number of neutrophils and NETs were quantified. Graphs depict the average and SEM for percent of neutrophils that formed total NETs (b), citrullinated NETs (c), and uncitrullinated NETs (d) for each condition with percent NETs for each stimulant compared between PAD2^+/+^ and PAD2^−/−^ mice. For all panels: *n* = 4; no comparisons were significant.

We then determined if PAD4 is required for the formation of NETs in response to the selected stimuli. Identical experiments as above were performed using bone marrow-derived neutrophils from PAD4^+/+^ and PAD4^−/−^ mice. As shown in Figures 3(a)–3(d), PAD4^−/−^ neutrophils generated almost no citrullinated NETs in response to any stimulus, with similar numbers of uncitrullinated NETs to PAD4^+/+^ neutrophils for all stimuli. The absence of citrullinated NETs led to a loss of total NETs in response to ionomycin, which primarily induces citrullinated NETs in mice. We then used identical methods and PAD2^−/−^ and PAD2^+/+^ mice to determine if PAD2 is required for NETosis. As shown in Figures 4(a)–4(d), PAD2^−/−^ mice showed no difference in the number of either citrullinated or uncitrullinated NETs induced by any stimulus. Repeating both the PAD4 and the PAD2 experiments using anti-citrullinated histone H4 showed the same findings: a loss of citrullinated NETs in the absence of PAD4 and no loss of NETs in the absence of PAD2 (Supplementary Figures 4 and 5).

## 4. Discussion

In this study, we quantified the formation of citrullinated and uncitrullinated NETs in response to ionomycin, PMA, MSU, and *C. albicans*. One conclusion from our studies is that human peripheral blood and murine bone marrow-derived neutrophils respond differently to stimuli. For example, ionomycin induced 66% of neutrophils to form NETs in mice and 28% in humans while PMA induced 9% of neutrophils to form NETs in mice and 77% in humans (Figures 1 and 2). Additionally, MSU was a strong inducer of NETs in human neutrophils and a variable inducer in murine neutrophils. Although some differences may be due to the location from which the neutrophils were purified (i.e., peripheral blood versus bone marrow) and thus maturation level, these findings suggest that NET production varies with the source of neutrophils, which may contribute to conflicting reports about the ability of different stimuli to induce NETs [21]. Other studies have identified a species-specific difference related to myeloperoxidase [44].

Regarding citrullination status, mice and humans often diverged again. For example, ionomycin-induced murine NETs were primarily citrullinated, whereas ionomycin-induced human NETs were a mix of citrullinated and uncitrullinated. *C. albicans* was a strong inducer of NETs in both mice and humans as previously shown [4, 23, 25, 45, 46] with primarily citrullinated NETs formed in humans and citrullinated and uncitrullinated in mice. Similarly, MSU induced mostly citrullinated NETs in humans and a mix in mice. In addition to highlighting the differences between mice and humans, our findings and the findings of others [25] do not support the theory that *C. albicans* or MSU inhibits citrullination [18]. In contrast and as expected [16, 17], for both humans and mice, PMA induced primarily uncitrullinated NETs, although some citrullinated NETs formed, particularly in humans. In neutrophils, PMA rapidly induces reactive oxygen species [17], which is required for PMA-induced NETs [38] and can inhibit PADs [47], perhaps explaining the relative lack of citrullination in addition to a reported role for PMA-induced protein kinase C alpha in PAD4 inhibition [16]. Of note, no stimulant in this study induced exclusively citrullinated or uncitrullinated NETs, a novel observation. It is possible that some citrullinated proteins were not detected by the F95 antibody and the immunofluorescence methodology, although F95 recognizes a variety of citrullinated proteins. We observed similar results using an anti-citrullinated histone H4 antibody (Supplementary Figures 2–5), although there were some differences, primarily in humans, potentially related to F95 detecting homocitrulline or the reactivity of anti-citrullinated histone H4 against only a single citrullinated protein.

Nonetheless, by quantifying both citrullinated and uncitrullinated NETs, we demonstrated that specific stimuli induce varying proportions of both citrullinated and uncitrullinated NETs in mice and humans, providing new insights into NETs. Although the citrullinated and uncitrullinated NETs could be categorized as resulting from LTH and NETosis, we did not observe that specific stimuli strictly induced either LTH with citrullination or NETosis without citrullination. Thus, the combination of stimulus and citrullination presence/absence may not be ideal for defining different NETs. Moreover, the generation of both citrullinated and uncitrullinated NETs in response to a single stimulus suggests that individual neutrophils may employ different pathways to generate NETs, sometimes involving citrullination and sometimes not. It will be important to further characterize the different mechanisms by which NETs with different characteristics form, since these differences may have important implications for autoimmune disease, especially rheumatoid arthritis with its citrulline-targeting autoantibodies.

Additionally, since we evaluated the requirement for PAD4 in both citrullinated and uncitrullinated NETs, whereas other groups evaluated either total or citrullinated NETs, we were able to demonstrate for apparently the first time that PAD4 is required for the production of citrullinated, but not uncitrullinated, NETs. These findings help to explain some of the discrepancies in the literature. Multiple studies have shown a requirement for PAD4 in NETosis [2, 28, 34–38]. However, many of these studies quantified citrullinated NETs. More recently, PAD4 was shown to be dispensable for *Klebsiella-*induced NET [39] and *C. albicans*-induced NET [25], in both cases with NETs detected primarily by DNA staining. Thus, some of the discrepancies among PAD4 studies may relate to whether only citrullinated NETs or total NETs were quantified. Indeed, PAD4 is required for histone citrullination induced by *Klebsiella* and *C. albicans* [25, 39]. Other discrepancies related to the role for PAD4 in NETosis may be due to methodology. For example, in a study that concludes that PAD4 is not required for ionomycin-induced NETs [25], the NETs were quantified by increased SYTOX fluorescence, not visualized NETs. Since ionomycin can form pores, perhaps those pores allowed SYTOX entry and DNA staining without NET formation. Our observation that PAD4 is required for the production of only citrullinated NETs also suggests that the formation of different NETs can have different requirements. Thus, it is important to assess both citrullinated and uncitrullinated NETs.

Finally, we evaluated PAD2 in NETosis. Previously, we demonstrated that PAD2 is not required for NETs induced by LPS and TNF*α* [28]. Here, we found that PAD2 is not required for the production or citrullination of murine NETs induced by ionomycin, MSU, PMA, or *C. albicans*, suggesting that PAD2 is not required for NETosis in general. Thus, although PAD2 is present in NETs [40], it is not required for their formation. This finding has interesting implications for rheumatoid arthritis. NETs have been hypothesized to be a significant source of citrullinated protein in rheumatoid arthritis [6, 7]. We, and others, have shown that PAD4 is required for the formation of citrullinated NETs [2, 28, 35]. However, in PAD4-deficient mice with inflammatory arthritis, total citrullination is not reduced in the serum, lung, or joint [28, 29, 48]. In contrast, PAD2 is required for a significant amount of citrullination in the joints of mice with inflammatory arthritis [28], but is not required to form citrullinated NETs. Although there are challenges related to the quantification of citrullination, taken together, these studies suggest that although NETs display citrullinated proteins targeted by anti-citrullinated protein antibodies [6], NETs may not be the main source of citrullinated proteins in rheumatoid arthritis. This theory is supported by the observations that PAD2 levels in synovial fluid correlate with total PAD activity and disease activity in rheumatoid arthritis [49], PAD2^−/−^ mice have less central nervous system citrullination in experimental autoimmune encephalomyelitis [41], and PAD2 has less restrictive substrate specificity than PAD4 [50].

## 5. Conclusion

This study demonstrates that various stimuli induce a mix of citrullinated and uncitrullinated NETs in mice and humans. Further, PAD4 is required for citrullinated NETs and PAD2 is not required for citrullinated or uncitrullinated NETs. Future studies are needed to further define different NETs, their mechanisms of formation, and their roles in the pathophysiology of autoimmune disease.

## Data Availability

The data used to support the findings of this study are available from the corresponding author upon request.

## Supplementary Materials

Supplementary Materials Supplementary Figure 1: Scanning electron micrographs of doNETs formed by PMA-stimulated human neutrophils. Supplementary Figure 2: Citrullinated NETs in human neutrophils detected by anti-citrullinated histone H4. Supplementary Figure 3: Citrullinated NETs in murine neutrophils detected by anti-citrullinated histone H4. Supplementary Figure 4: PAD4 is required for the formation of citrullinated NETs in murine neutrophils as detected by anti-citrullinated histone H4. Supplementary Figure 5: PAD2 is not required for the formation of citrullinated NETs in murine neutrophils as detected by anti-citrullinated histone H4.
