# Supplementary material for: Insight into Neutrophil Extracellular Traps through Systematic Evaluation of Citrullination and Peptidylarginine Deiminases
Source: J Immunol Res. 2019 Mar 12;2019:2160192. doi: 10.1155/2019/2160192 (PMC6434303; doi:10.1155/2019/2160192)
Supplement: Supplementary Materials — Supplementary Figure 1: Scanning electron micrographs of doNETs formed by PMA-stimulated human neutrophils. Supplementary Figure 2: Citrullinated NETs in human neutrophils detected by anti-citrullinated histone H4. Supplementary Figure 3: Citrullinated NETs in murine neutrophils detected by anti-citrullinated histone H4. Supplementary Figure 4: PAD4 is required for the formation of citrullinated NETs in murine neutrophils as detected by anti-citrullinated histone H4. Supplementary Figure 5: PAD2 is not required for the formation of citrullinated NETs in murine neutrophils as detected by anti-citrullinated histone H4. [file 2160192.f1.pdf]

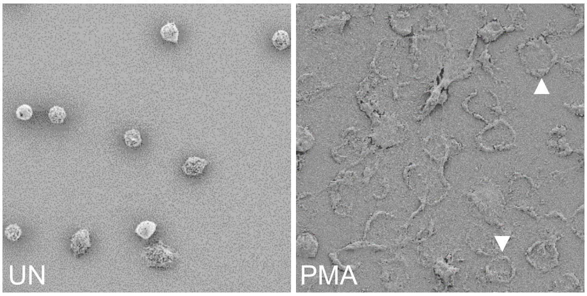

Supplementary Figure 1: Scanning electron micrographs of doNETs formed by PMA-stimulated human neutrophils. Treated neutrophils were washed with PBS, fixed overnight (4% formaldehyde and 1% glutaraldehyde in PBS), washed with PBS, treated with 1% osmium tetroxide, washed again, dehydrated via ethanol washes and critical point drying, coated with platinum, and imaged in a scanning electron microscope (LEO 1530-2 FESEM) at 3kV with SmartSEM software (Zeiss, Oberkochen, Germany). Representative images at 2,000x of untreated (UN) or PMA treated neutrophils are shown.

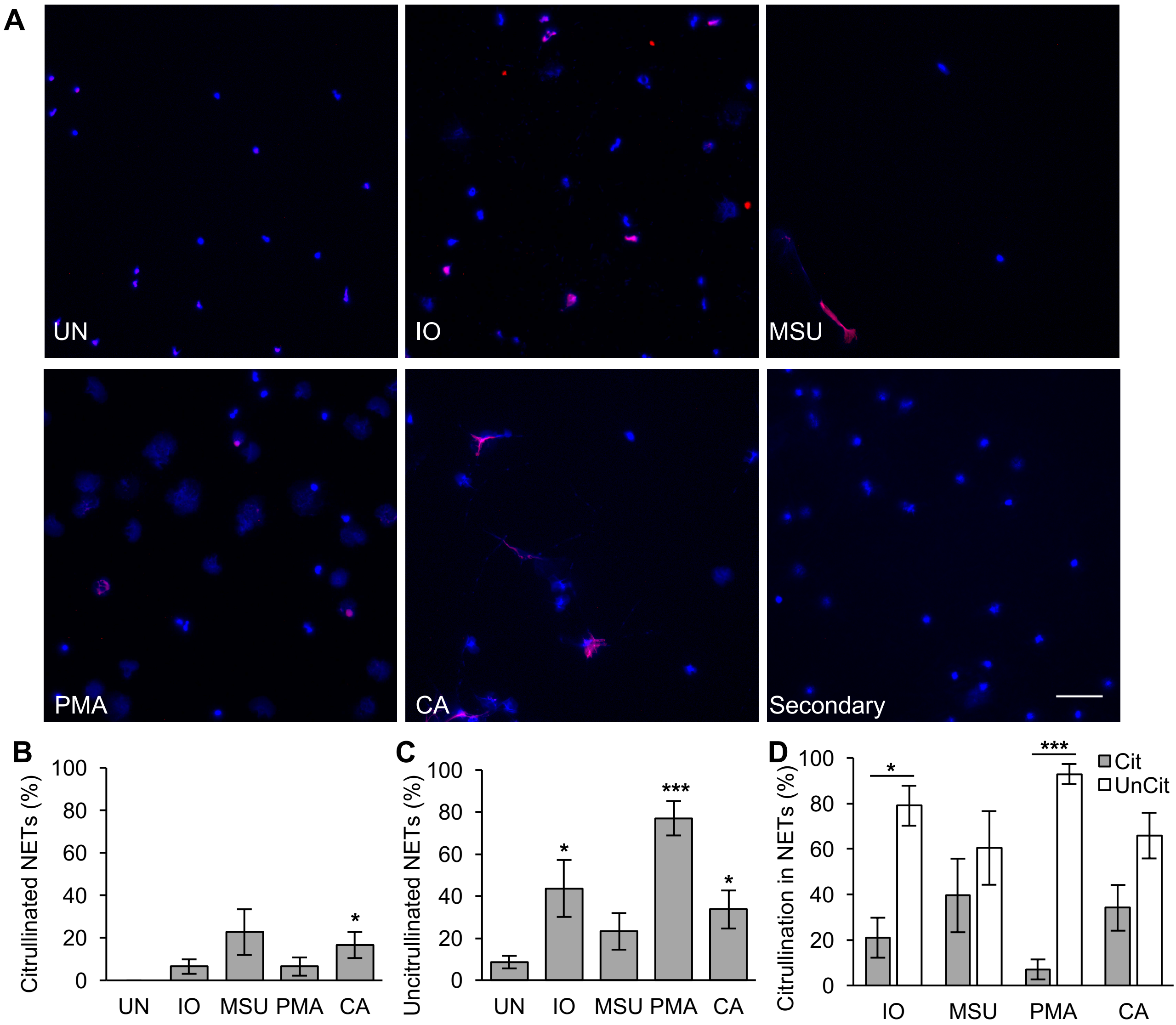

Supplementary Figure 2: Citrullinated NETs in human neutrophils detected by anti-citrullinated histone H4. Human neutrophils were left untreated (UN) or were treated with ionomycin (IO), MSU, PMA, or *C. albicans* (CA), fixed, and stained with DAPI (blue) and anti-histone 4 citrulline 3 antibody (pink). Image labeled "Secondary" was created by stimulating neutrophils with *C. albicans* and staining without the histone 4 citrulline 3 primary antibody and only the anti-rabbit IgG TRITC secondary antibody as a negative control. (A) Representative images at 400x, scale bar = 50μM. The number of neutrophils and NETs were quantified. Graphs depict the average and SEM for percent of neutrophils that formed citrullinated NETs (B) and uncitrullinated NETs (C) for each condition with percent NETs for each stimulant compared to untreated. (D) The percent of citrullinated versus uncitrullinated NETs was compared for each stimulus with average and SEM graphed. For all panels, n=6, *p<0.05, ***p<0.001.

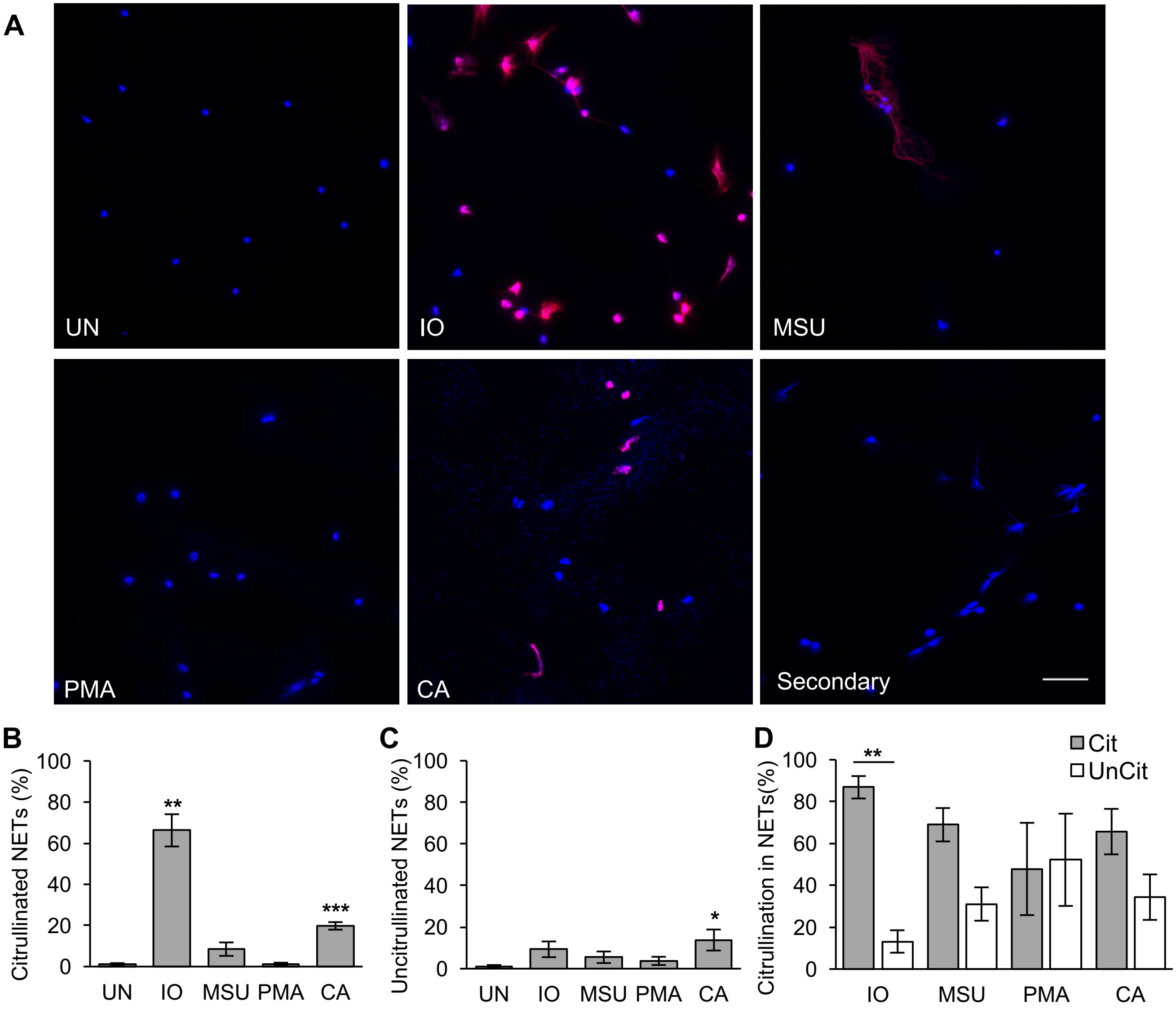

Supplementary Figure 3: Citrullinated NETs in murine neutrophils detected by anti-citrullinated histone H4. Mouse neutrophils were left untreated (UN) or were treated with ionomycin (IO), MSU, PMA, and *C. albicans* (CA), fixed, and stained with DAPI (blue) and anti-histone 4 citrulline 3 antibody (pink). Image labeled “Secondary” was created by stimulating neutrophils with ionomycin and staining without the histone 4 citrulline 3 primary antibody and only the anti-rabbit IgG TRITC secondary antibody as a negative control. (A) Representative images at 400x, scale bar = 50μM. The number of neutrophils and NETs were quantified. Graphs depict the average and SEM for percent of neutrophils that formed citrullinated NETs (B) and uncitrullinated NETs (C) for each condition with percent NETs for each stimulant compared to untreated. (D) The percent of citrullinated versus uncitrullinated NETs was compared for each stimulus with average and SEM graphed. For all panels, n=5, *$p<0.05$, **$p<0.01$, ***$p<0.001$.

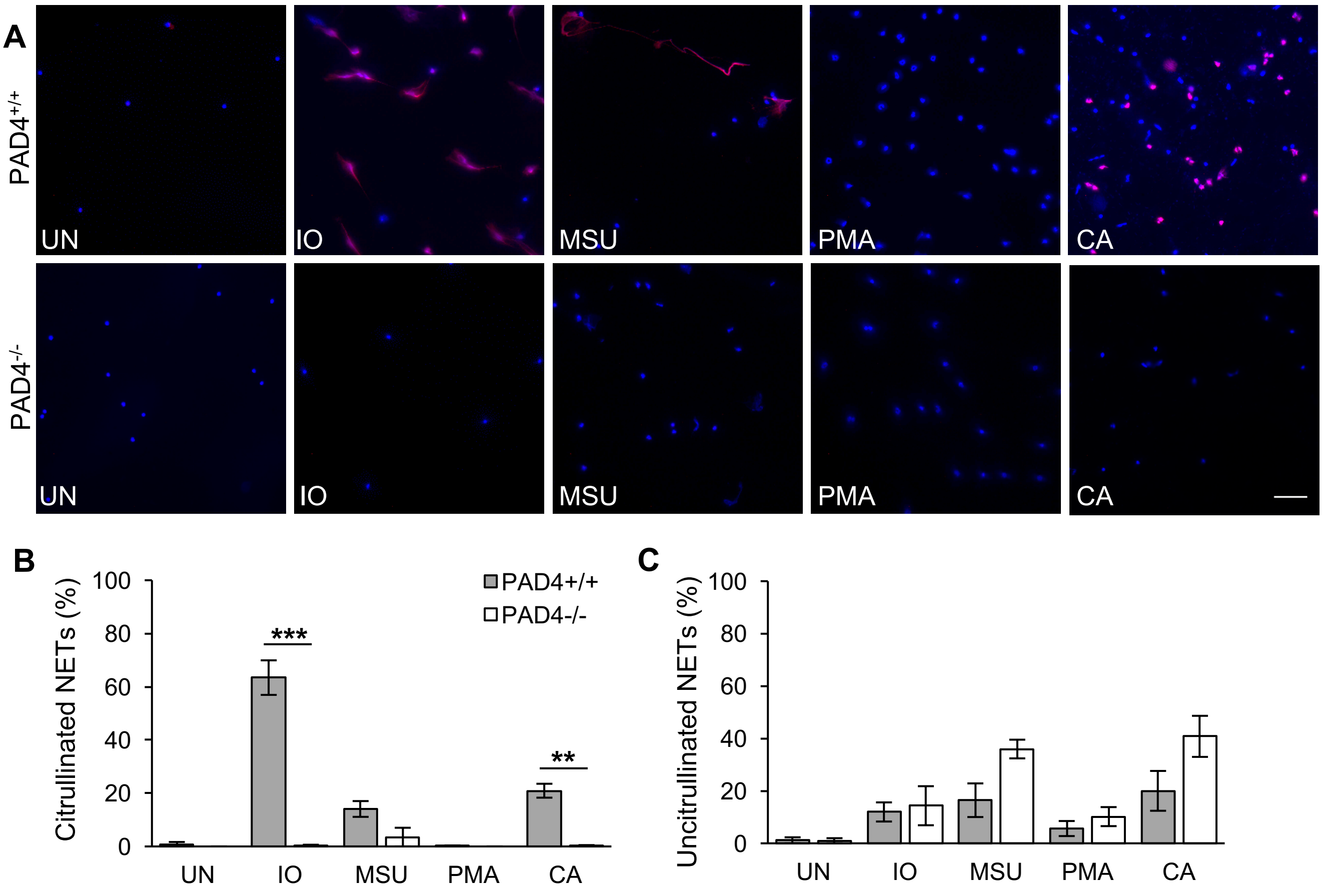

Supplementary Figure 4: PAD4 is required for the formation of citrullinated NETs in murine neutrophils as detected by anti-citrullinated histone H4. Bone marrow neutrophils from $PAD4^{+/+}$ and $PAD4^{-/-}$ mice were left untreated (UN) or were treated with ionomycin (IO), MSU, PMA, and *C. albicans* (CA), fixed, and stained with DAPI (blue) and anti-histone 4 citrulline 3 antibody (pink). (A) Representative images at 400x, scale bar = 50μM. The number of neutrophils and NETs were quantified. Graphs depict the average and SEM for percent of neutrophils that formed citrullinated NETs (B) and uncitrullinated NETs (C) for each condition with percent NETs for each stimulant compared between $PAD4^{+/+}$ and $PAD4^{-/-}$ mice. For all panels, n=3, $^{**}p<0.01$, $^{***}p<0.001$.

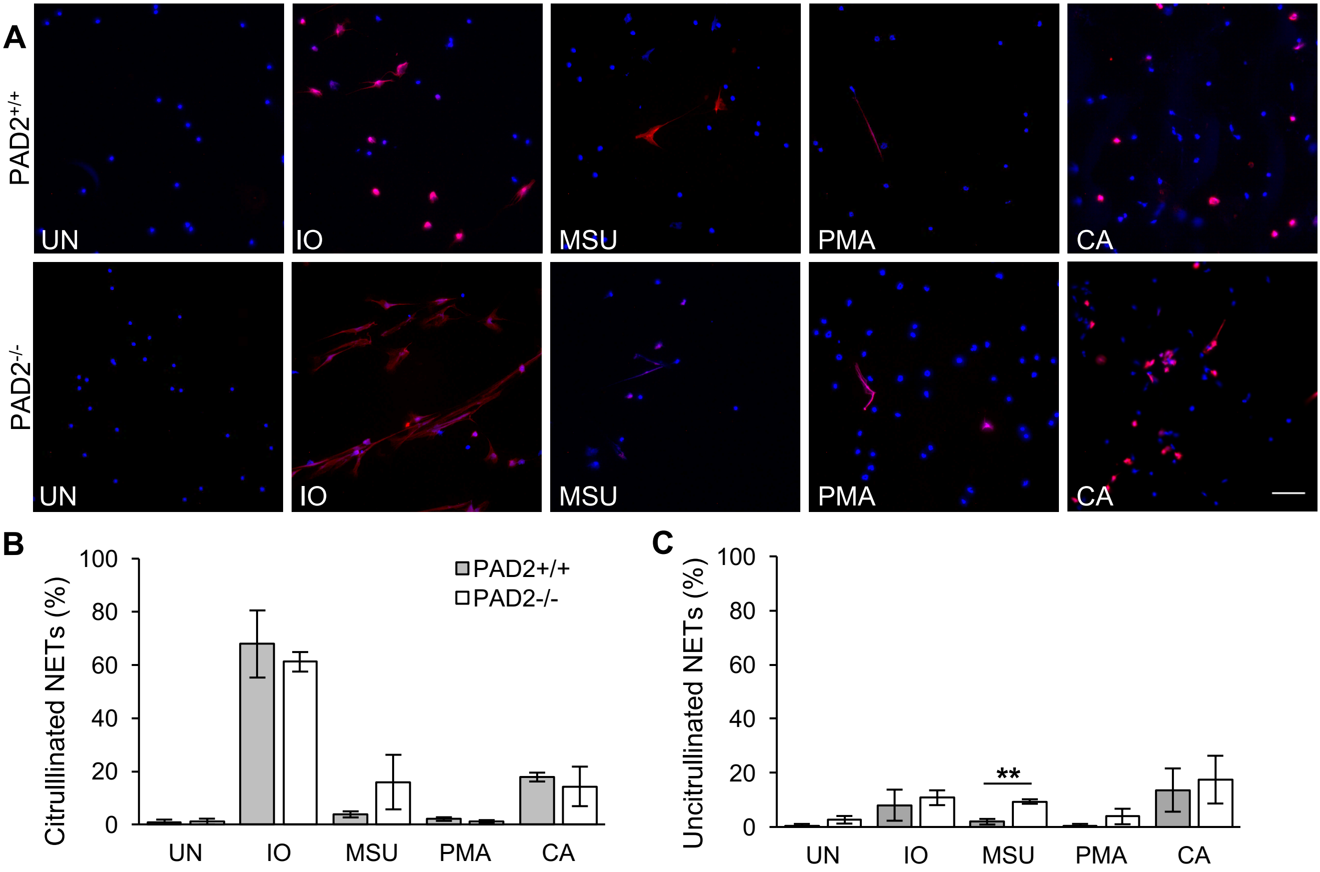

Supplementary Figure 5: PAD2 is not required for the formation of citrullinated NETs in murine neutrophils as detected by anti-citrullinated histone H4. Bone marrow neutrophils from $PAD2^{+/+}$ and $PAD2^{-/-}$ mice were left untreated (UN) or were treated with ionomycin (IO), MSU, PMA, and *C. albicans* (CA), fixed, and stained with DAPI (blue) and anti-histone 4 citrulline 3 antibody (pink). (A) Representative images at 400x, scale bar = 50μM. The number of neutrophils and NETs were quantified. Graphs depict the average and SEM for percent of neutrophils that formed citrullinated NETs (B) and uncitrullinated NETs (C) for each condition with percent NETs for each stimulant compared between $PAD2^{+/+}$ and $PAD2^{-/-}$ mice. For all panels, n=3, $^{**}p<0.01$.
